# Development of immortalized Hertwig’s epithelial root sheath cell lines for cementum and dentin regeneration

**DOI:** 10.1186/s13287-018-1106-8

**Published:** 2019-01-03

**Authors:** Xuebing Li, Sicheng Zhang, Zirui Zhang, Weihua Guo, Guoqing Chen, Weidong Tian

**Affiliations:** 1 State Key Laboratory of Oral Disease, West China Hospital of Stomatology, Sichuan University, Chengdu, China; 2 National Engineering Laboratory for Oral Regenerative Medicine, West China Hospital of Stomatology, Sichuan University, Chengdu, China; 3 National Clinical Research Center for Oral Diseases, West China Hospital of Stomatology, Sichuan University, Chengdu, China; 4 Department of Oral and Maxillofacial Surgery, West China Hospital of Stomatology, Sichuan University, Chengdu, China; 5 Department of Pediatric, West China Hospital of Stomatology, Sichuan University, Chengdu, China; 6 West China School of Public Health, Sichuan University, Chengdu, China

**Keywords:** Hertwig’s epithelial root sheath, Cementum, Dentin, Tooth regeneration

## Abstract

**Background:**

Hertwig’s epithelial root sheath (HERS) is important in guiding tooth root formation by differentiating into cementoblasts through epithelial–mesenchymal transition (EMT) and inducing odontoblastic differentiation of dental papilla through epithelial–mesenchymal interaction (EMI) during the tooth root development. Thus, HERS cells are critical for cementum and dentin formation and might be a potential cell source to achieve tooth root regeneration. However, limited availability and lifespan of primary HERS cells may represent an obstacle for biological investigation and therapeutic use of tooth tissue engineering. Therefore, we constructed, characterized, and tested the functionality of immortalized cell lines in order to produce a more readily available alternative to HERS cells.

**Methods:**

Primary HERS cells were immortalized via infection with lentivirus vector containing the gene encoding simian virus 40 Large T Antigen (SV40LT). Immortalized HERS cell subclones were isolated using a limiting dilution method, and subclones named HERS-H1 and HERS-C2 cells were isolated. The characteristics of HERS-H1 and HERS-C2 cells, including cell proliferation, ability of epithelial–mesenchymal transformation and epithelial–mesenchymal interaction, were determined by CCK-8 assay, immunofluorescence staining, and real-time PCR. The cell differentiation into cementoblast-like cells or periodontal fibroblast-like cells was confirmed in vivo. And the inductive influence of the cell lines on dental papilla cells (DPCs) was also confirmed in vivo.

**Results:**

HERS-H1 and HERS-C2 cells share some common features with primary HERS cells such as epithelial-like morphology, positive expression of CK14, E-Cadherin, and Vimentin, and undergoing EMT in response to TGF-beta. HERS-C2 cells showed the EMT characteristics and could differentiate into cementum-forming cells in vitro and generate cementum-like tissue in vivo*.* HERS-H1 could induce the differentiation of DPCs into odontoblasts in vitro and generation of dentin-like tissue in vivo.

**Conclusions:**

We successfully isolated and characterized novel cell lines representing two key features of HERS cells during the tooth root development and which were useful substitutes for primary HERS cells, thereby providing a biologically relevant, unlimited cell source for studies on cell biology, developmental biology, and tooth root regeneration.

**Electronic supplementary material:**

The online version of this article (10.1186/s13287-018-1106-8) contains supplementary material, which is available to authorized users.

## Background

Tooth loss is a common and frequent situation resulted from numerous pathologies such as trauma, endodontic complications, and periodontal and carious diseases. Due to the limitations of traditional artificial repair of tooth loss, stem cell-based tissue engineering is considered a valid therapeutic approach to construct a tooth with normal physiological functions [1]. There have been various kinds of mesenchymal stem cells for generating bioengineered tooth tissues, such as dental pulp cells, dental follicle cells (DFCs), and bone marrow stromal cells (BMSCs) [2–4]. From a developmental point of view, the development and growth of a functional tooth are complex processes, in which reciprocal interaction between mesenchymal and epithelial cells plays an important role [1]. Although significant progress has been made with mesenchymal cells in tooth tissue engineering, there is little information available for dental epithelial stem cells.

The Hertwig’s epithelial root sheath (HERS) is a bilayer epithelial sheath originated from the apical region of the enamel organ [5]. As for the process of tooth root development, HERS plays an important role in guiding root formation and determining the size, shape, and number of tooth roots [6]. During the tooth root development, HERS cells can differentiate into cementum-forming cells via the process of epithelial–mesenchymal transition (EMT) [7, 8]. With the elongation of HERS, the dental papilla comes to contact with HERS and differentiate into odontoblasts, which secrete dentin of the root. Moreover, the HERS also produces growth factors that contribute to the induction of odontoblast differentiation [6]. If the structure of HERS is disturbed prematurely, the differentiation of root odontoblasts is compromised [9]. Thus, it is confirmed that HERS functions as a development center to guide root formation. As for the mechanism involved in the HERS cells or odontoblast differentiation, our and other previous studies had shown that TGF-β1 or FGF2 could trigger the EMT of HERS cells, in which PI3K/AKT and MAPK/ERK signaling was involved [7, 8]. BMP/TGF-β signaling pathway was mainly discussed in the differentiation of odontoblasts during tooth root development [10–13]. However, HERS is a fleeting, transient structure assembled in the early period of root formation and elongation, which makes it difficult to obtain enough primary cells for biological and preclinical studies. With the root formation, HERS cells fenestrated and reduced to epithelial rests of Malassez (ERM) after root dentin deposition. There have been studies speculated that ERM could develop into cementum-forming cells and reported that immortalized cell lines had already been established [7, 14, 15]. Nevertheless, these cells are terminal products of HERS, which may not completely reflect the original characteristics of HERS cells in developing tooth. Therefore, most aspects of the biology of HERS cells are still obscure because of the lack of efficient methods to isolate stable phenotype and enough primary cells.

As mentioned above, the HERS cells play a critical role during the tooth root development via contributing to cell differentiation. However, there have been few reports concerned with the use of HERS cells in stem cell-based approaches of tooth tissue engineering. In this context, HERS cells might be a potential source for the construction of tooth root. In this study, we isolated primary HERS cells from SD rat and successfully established two immortalized cell lines with the characteristics of undergoing EMT and conducting epithelial–mesenchymal interaction (EMI) respectively. Importantly, we certified their potential of differentiation into cementoblasts and the generation of cementum-like tissue in vivo, and induction of differentiation of dental papilla cells (DPCs) and the formation of dentin-like tissue were also evaluated in vivo. Our data indicates that immortalized HERS cells can provide substitutes for primary cells in biologically relevant applications in the fields of tooth root regeneration.

## Materials and methods

All experiments were conducted in accordance with a protocol approved by the Committee of Ethics of West China Hospital of Stomatology of Sichuan University (NO. WCHSIRB-D-2018-100).

### Primary cell isolation and lentiviral transduction

**Fig. 1:**
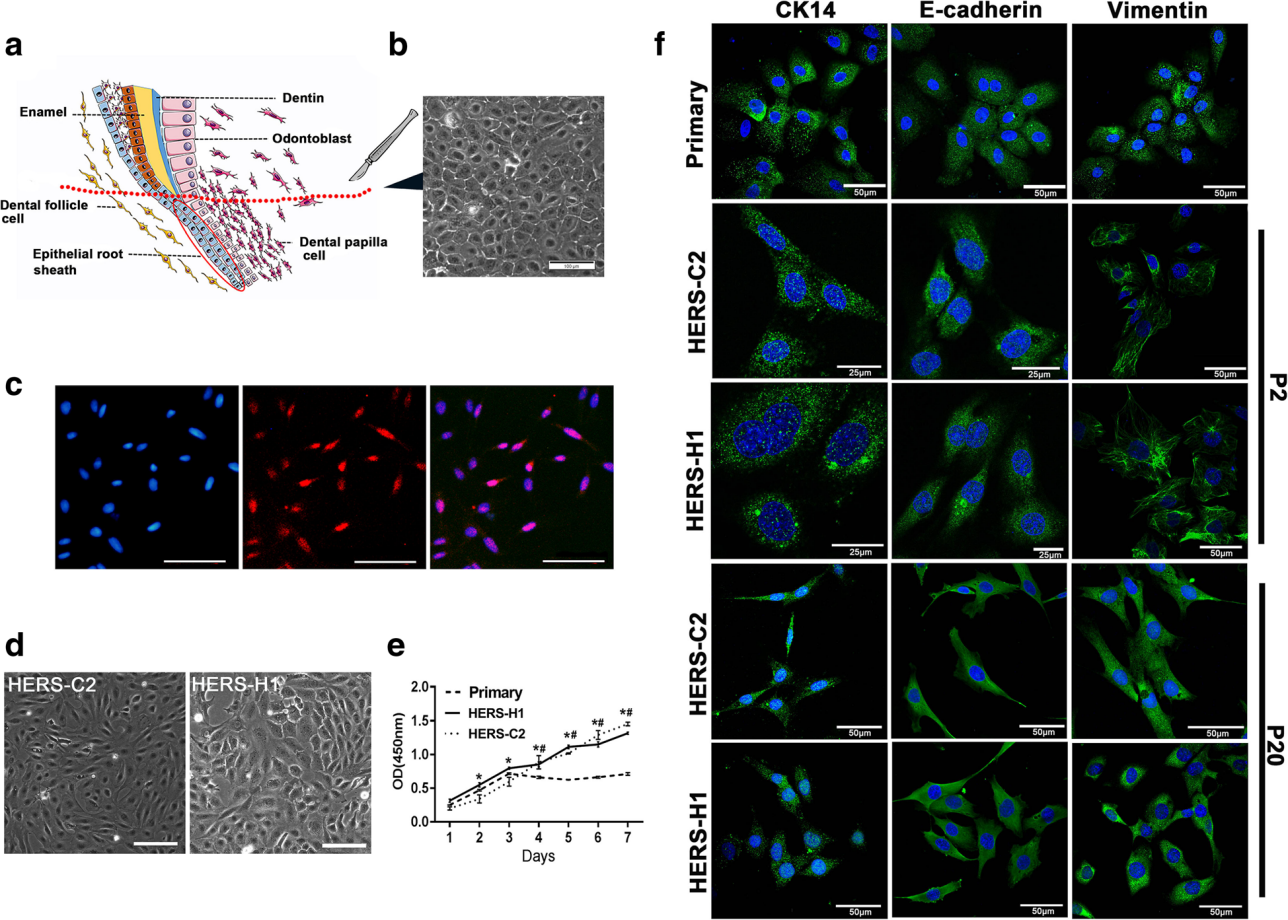
Establishment of the immortalized HERS cell lines and they expressed markers specific to HERS cells. **a** Isolation of HERS cells from PN8 rat first mandibular molars. **b** The primary HERS cells showed a typical cobblestone-like appearance. **c** SV40 T-Ag was present in the nucleus of the immortalized HERS cells. **d** Compared with the primary cells, the two cell lines showed an epithelial morphology and are a little bit elongated. **e** CCK-8 assay was used to compare the proliferation rate of the primary cells, HERS-H1 and HERS-C2. **f** The primary HERS cells expressed both epithelial cell markers CK14, E-cadherin, and mesenchymal cell markers vimentin. The immortalized cells HERS-C2 and HERS-H1 also expressed CK14, E-cadherin, and vimentin stably at least from passage 2 to passage 20. Scale bars are shown. (*P < 0.05, HERS-H1 vs. primary HERS cells; #*P* < 0.05, HERS-C2 vs. primary HERS cells, Scale bar: 100 μm in **b** and **d**)

The primary DPCs were obtained and cultured as previously described [16]. For the primary HERS cell isolation and culture, as shown in Fig. 1a, Hertwig’s epithelial root sheath of the tooth apical region of the mandibular first molars at PN8 was dissected, the tissues were dissociated with 2.4 U/mL dispase (Sigma-Aldrich, St Louis, MO) and 625 U/mL collagenase (Sigma-Aldrich, St Louis, MO), which was cultured with epithelial cell medium (ScienCell, CA, USA) consisting of basal medium, 2% fetal bovine serum, 1% epithelial cell growth supplement, and 1% penicillin/streptomycin solution [8, 17]. Purified primary HERS cells at passage 1 were transfected with lentiviral vector (pGMLV-SV40T-PURO, Genomeditech, Shanghai, China), which encoded simian virus 40 Large T Antigen (SV40 LT) and a puromycin resistance gene. After transfection, the cells were selected by supplementing the media with 2 μg/ml puromycin (Sigma-Aldrich, St Louis, MO). Using the limited dilution method, cells were isolated as single cells and cultured to expand the selected clones.

### Cell proliferation assay

Cell proliferation was detected using Cell Counting Kit-8 (Dojindo, Japan) according to the manufacturer’s instructions. Cells at passage 2 were plated at a density of 5000 cells per well in 96-well plates with the complete media. The original cultivation medium was replaced by 120 μl fresh medium containing 12 μl CCK-8 at the same time of consecutive 7 days. After 2 h incubation at 37 °C, the absorbance of the solution was determined with a spectrophotometer (Thermo Scientific, USA) at 450 nm. Five independent experiments were performed in each group.

### Real-time PCR

Cells at passage 4 were in an undifferentiated status and treated with 10 ng/ml TGF-β1. RNA was isolated from cells using RNAiso Plus (Takara, Japan) according to the manufacturer’s protocol. Four micrograms of RNA were reverse transcribed to cDNA with Revert Aid First Strand cDNA Synthesis Kit Transcription Kit (Thermo Scientific, USA). Real-time RT-PCR was conducted as previously described using SYBR Premix Ex Taq (TaKaRa Biotechnology, Japan) [16]. The reaction was performed with QuantStudio 6 Flex (Applied Biosystems, Foster City, CA, USA). All of the operating procedures followed the manufacturer’s protocol. The PCR primer sequences are shown in Additional file 1: Table S1. All experiments were performed in triplicates and repeated three times.

### Immunofluorescence and immunohistochemical staining

Immunofluorescence and immunohistochemical staining was performed as previously described [8, 18]. Cells or tissue sections were examined under fluorescence confocal microscope (Olympus FV1000, Japan) or microscope (Olympus, IX73, Japan). The primary and secondary antibodies and their dilutions used in this study were as follows: mouse anti-CK14 (1:200, MAB3232, Millipore, CA, USA), mouse anti-vimentin (1:200, sc-6260, Santa Cruz, CA, USA), rat anti-E-cadherin (1:100, ab11512, Abcam, MA, USA), rabbit anti-SV40 Large T antigen (1:200, #15729, Cell Signaling Technology, CA, USA), goat anti-DMP1 (1:200, sc-6551, Santa Cruz, CA, USA), rabbit anti-BSP (1:200, D261488, Sangon Biotech, Shanghai, China), rabbit anti-OCN (1:200, AP2002a 614487, Zenbio, Chengdu, China), rabbit anti-Periostin (1:200, sc-67233, Santa Cruz, CA, USA), rabbit anti-DSP (1:200, sc-33587, Santa Cruz, CA, USA), Alexa FluoR 488 Goat anti Mouse (1:500, A11001, Invitrogen, Eugene, OR, USA), Alexa FluoR 488 Goat anti Rabbit (1:500, A11008, Invitrogen, Eugene, OR, USA), Alexa FluoR 555 Goat anti Rabbit (1:500, A11008, Invitrogen, Eugene, OR, USA), donkey anti-goat IgG-HRP (1:500, sc-2020, Santa Cruz, CA, USA), and goat anti-rabbit IgG-HRP (1:500, ZB-2301, ZSGB-BIO, Beijing, China). Negative controls were carried out by substituting a normal IgG for the primary antibody.

### Mineralization assay

Cells were cultured in osteo-inductive medium with or without 10 ng/mL TGF-β1. After 3 and 7 days culture, Total RNA was isolated from cells for quantitative real-time PCR analysis**.** After 4 weeks culture, the cells were fixed with 4% paraformaldehyde and stained with 0.1% Alizarin red S (Sigma-Aldrich, St Louis, MO) for 30 min at room temperature. Mineralized bone nodules were destained with 10% cetylpyridinium chloride in double distilled water, and the calcium concentration was determined by absorbance measurements at 405 nm. All experiments were performed at least in triplicate.

### Conditioned medium preparation and treatment

The conditioned medium (CM) of the two cell lines was prepared as described previously [19]. The DPCs were cultured with HERS-H1 CM or HERS-C2 CM and refreshed every 24 h. At a different time point, the cells co-cultured with CM were collected to perform real-time PCR. All experiments were performed at least in triplicate.

### In vivo transplantation of cultured cells

HERS-C2 and HERS-H1 cells (treated with 10 ng/mL TGF-β1 for 48 h) alone or combined with DPCs (HERS-C2 or HERS-H1: DPCs = 1:2, a total of approximately 2.0 × 10^6^ cells) were mixed with 40 mg hydroxyapatite/tricalcium phosphate (HA/TCP) ceramic powder (The Engineering Research Center in Biomaterials, Sichuan University), and two pellets of HA/TCP with adherent cells was then transplanted into left renal capsule of 8-week female SD rats (Dashuo Experimental Animal Co. Ltd., Chengdu, China). The 24 rats were randomly divided into six groups: Control group (HA/TCP without cells), HERS-C2 group (HERS-C2 cells mixed with HA/TCP), HERS-H1 group (HERS-H1 cells mixed with HA/TCP), DPC group (DPCs mixed with HA/TCP), HERS-C2 combined with DPC group (HERS-C2 combined with DPCs mixed with HA/TCP), and HERS-H1 combined with DPC group (HERS-H1 combined with DPCs mixed with HA/TCP). There were at least four replicates in each group. All animals were maintained under standardized conditions with the temperature of 21 °C and a 12-h light cycle and had free access to food and water. The grafts were obtained at 8 weeks post-operatively, fixed with 4% paraformaldehyde, decalcified with buffered 10% EDTA, and then embedded in paraffin for further experiments.

### Tumorigenicity assay

To determine whether the HERS-C2 and HERS-H1 cells were tumorigenic, 1.0 × 10^5^ cells combined with Matrigel (BD Biosciences, USA) were subcutaneously injected into female 4–5-week-old balb/c nude mice (18–20 g) (Vital River Laboratory Animal Technology Co., Ltd., Beijing, China). The tumorigenic and immortalized oral squamous carcinoma SCC-25 cells were used as positive control. The mice were randomly assigned to five groups (*n* = 4 mice/group): control group (sham); primary HERS cells group; HERS-C2 cells group, HERS-H1 cells group, and SCC25 cells group. Animals were maintained under specific pathogen-free (SPF) facility with access ad libitum to food and fresh water and were monitored for tumor formation. Mice were sacrificed after 4 weeks culture and transplanted parts were obtained for further study.

### Microarrays and gene expression analysis

Agilent gene expression arrays (Kangchen Bio-tech Inc., Shanghai. China) were used to investigate the global expression profile of primary HERS cells, HERS-H1 and HERS-C2 cells (*n* = 3). The array represented more than 41,000 transcripts. The records of microarrays data have been approved and assigned the accession number GEO: GSE109622. The microarray datasets were normalized in GeneSpring GX v12.1 software package (Agilent Technologies). A *p* value of less than 0.01 and a mean expression change of greater than twofold was considered statistically significant and these genes were used for further analysis. Gene ontology and signaling pathway analysis of significantly different genes were analyzed using the DAVID online analysis tool (http://david.abcc.ncifcrf.gov/).

### Statistical analysis

All data were expressed as mean value ± standard deviation for each group. Statistical significance was assessed by using Student’s *t* test for two groups or analysis of variance (Tukey’s test) for multiple groups. *P* < 0.05 was considered as statistically significant.

## Results

### Phenotypic characteristics of two immortalized HERS cell lines HERS-C2 and HERS-H1

The primary HERS cells showed a cobblestone appearance (Fig. 1b). These cells were transfected with lentiviral vector encoding SV40 LT and selected with puromycin. Immunofluorescence detection showed the immortalized cell lines were positive expression of SV40 T-Ag in the nuclear (Fig. 1c). By selection for clonogenic cells, a total of 68 clones were selected which could be cultured for more than 50 passages. Among them, the two cell lines named HERS-H1 and HERS-C2 were used in present study for their unique features. These two type cells, showing a cobblestone-like morphology (Fig. 1d), were adherent and had a high proliferation capacity, with a doubling time of about 24 h (Fig. 1e). All the cells were positive for epithelial markers cytokeratin 14 (CK14) and E-cadherin and mesenchymal marker vimentin, which suggested the cells maintained the characteristics of both epithelial and mesenchymal cells. HERS-C2 and HERS-H1 maintained the expression of HERS cells markers at least 20 passages (Fig. 1f).

To determine if the immortalized HERS-C2 and HERS-H1 cells were tumorigenic, they were injected subcutaneously into immunodeficient athymic mice. No tumor formation was observed after 4 weeks, whereas the SCC-25 tumor cells formed large tumors within much shorter time (Additional file 2: Figure S1a and S1b).

### EMT characteristics of HERS-C2 and HERS-H1 cells

**Fig. 2:**
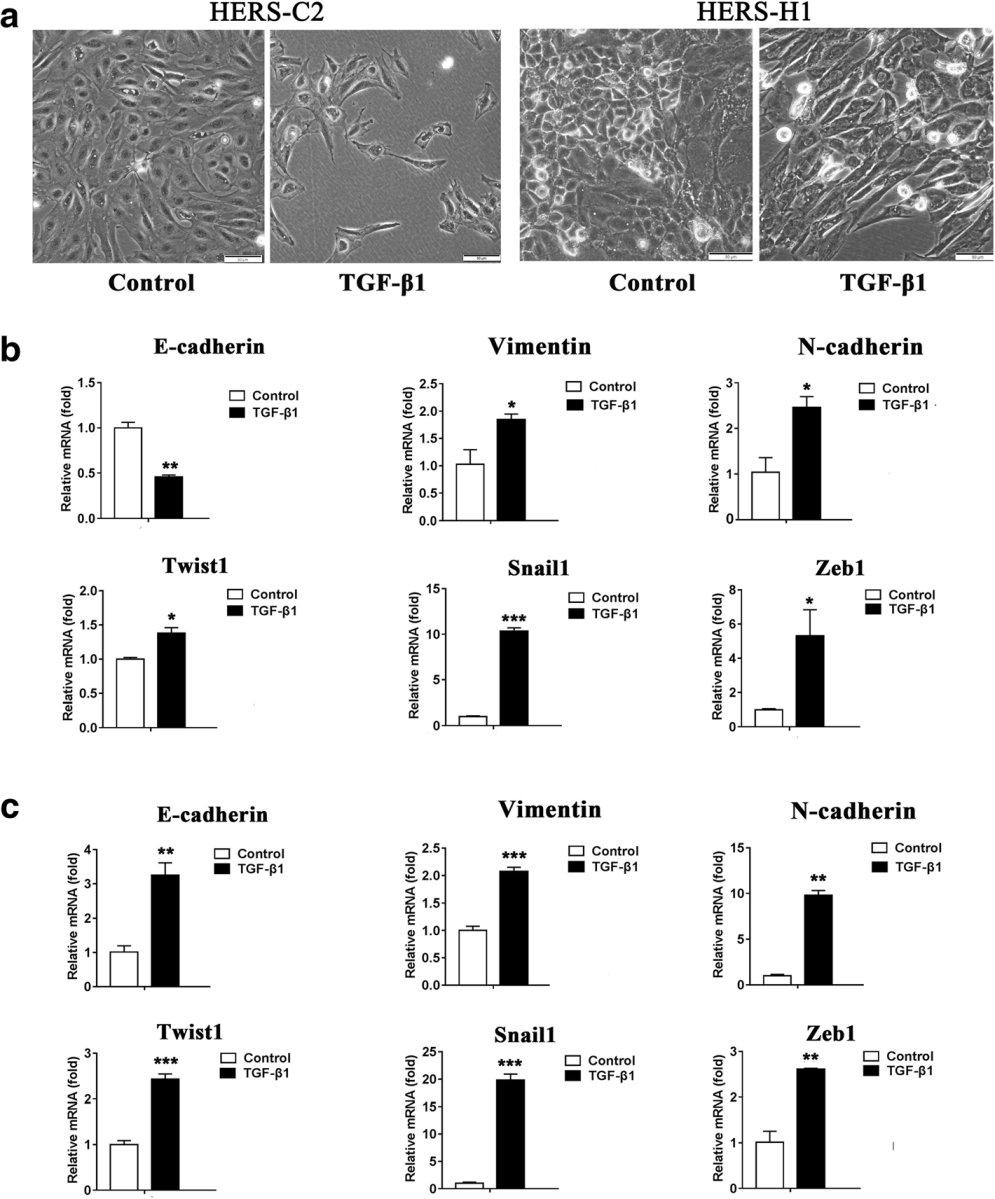
HERS-C2 and HERS-H1 underwent EMT induced by TGF-β1. **a** With the control culture media, HERS-C2 and HERS-H1 showed the cubostone-like morphology of epithelial cells. After 7 days induction by TGF-β1, HERS-C2 and HERS-H1 became elongated. Scale bars: 50 μm. **b**, **c** Expression of EMT markers, such as E-cadherin, Vimentin, N-cadherin, Twist1, Snail1, and Zeb1 was examined by real-time RT-PCR in **b** HERS-C2 cells and **c** HERS-H1 cells after 3 days treatment with TGF-β1. (*P < 0.05; **P < 0.01; ***P < 0.001 vs. control)

In previous studies, primary HERS cells could undergo EMT and acquire a mesenchymal phenotype with the induction of TGF-β1 [7, 8]. To investigate the properties of EMT of the immortalized cell lines, HERS-C2 and HERS-H1 were treated with TGF-β1 for 3 days and 7 days. TGF-β1 treatment triggered a partial morphological alteration of HERS-C2 and HERS-H1, from typical cobblestone-like epithelial cells to spindle-shape mesenchymal-like cells (Fig. 2a). After 3 days treatment, the expression of epithelial-associated gene E-cadherin was decreased while the mesenchymal-associated genes including vimentin and N-cadherin were increased in HERS-C2 cells (Fig. 2b**)**. The transcription factors twist1, snail1, and zeb1 were upregulated (Fig. 2b). As for HERS-H1 cells, the expression of epithelial-associated gene E-cadherin was upregulated at 3 days after TGF-β1 treatment (Fig. 2c) and downregulated until 7 days after TGF-β1 treatment (Additional file 3: Figure S2**)**; the expression levels of vimentin and N-cadherin were increased after 3 days or 7 days treatment (Fig. 2c and Additional file 3: Figure S2). The transcription factors twist1, snail1, and zeb1 were upregulated (Fig. 2c). These data suggested that immortalized HERS cells could respond to TGF-β1 and acquire mesenchymal phenotypes through EMT.

### Potential of cementoblastic differentiation of HERS-C2 and HERS-H1 cells in vitro and in vivo

**Fig. 3:**
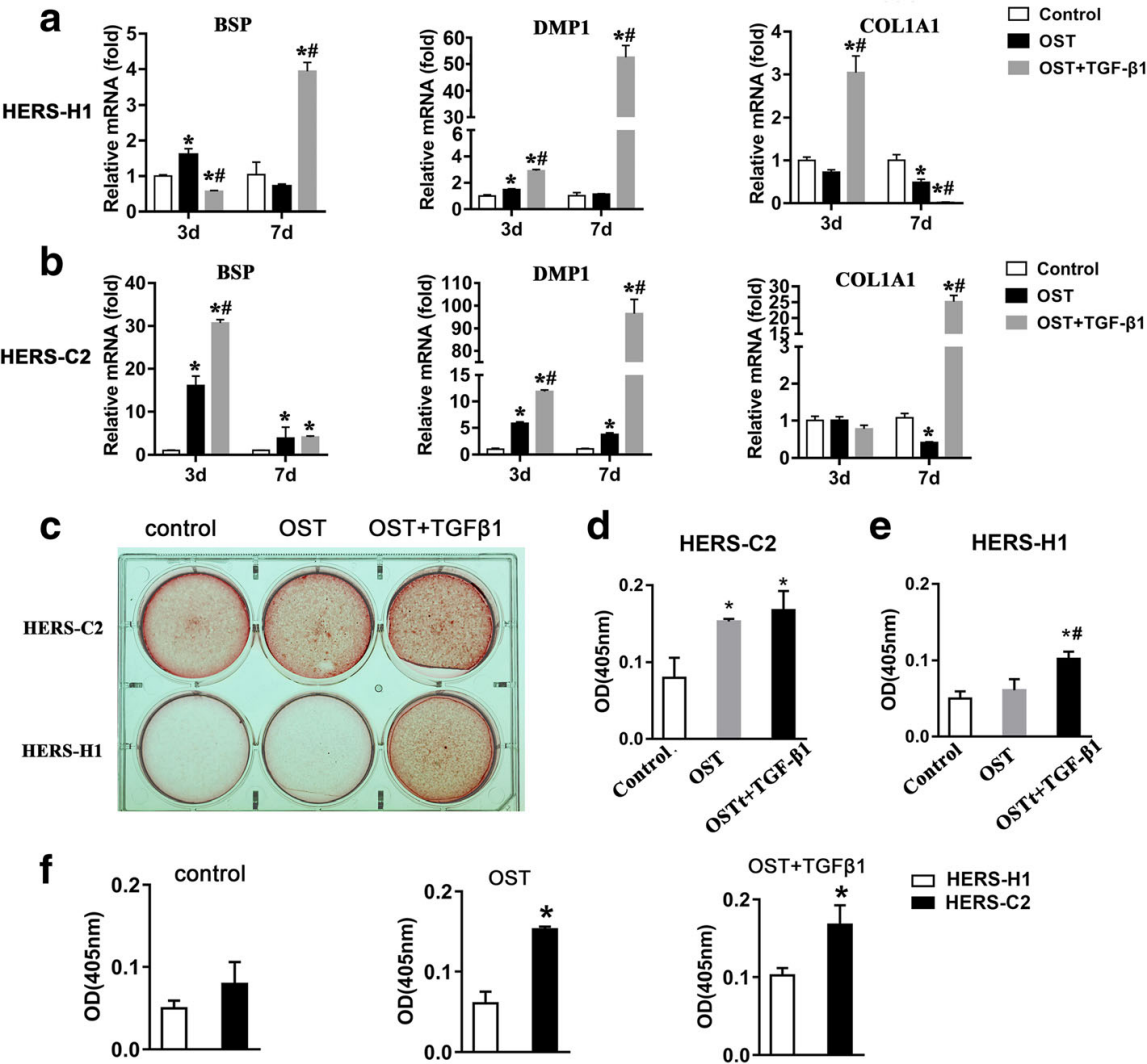
Cementogenetic differentiation potential of the two cell lines. **a**, **b** The expression of cementoblasts markers was examined by real-time RT-PCR in the primary, HERS-H1, and HERS-C2 cells after 3 and 7 days culture of osteo-medium and combined treatment with TGF-β1 and osteo-medium. **c** Representative images of Alizarin Red S staining indicated the potential of cemento-differentiation of HERS-H1 and HERS-C2 cells. **d**, **e**, **f** Quantitative analyses of calcium mineralization in **d** HERS-C2 cells and **f** HERS-H1 cells; **f** comparison between HERS-H1 and HERS-C2 cells. (*P < 0.05 vs. control group in **a**, **b**, **d**, **e**; #*P* < 0.05 vs. osteo-induction group; *P < 0.05 vs. HERS-H1 in **f**)

To address the potential of the two cell lines to undergo cementoblastic differentiation, we cultured the cells with osteo-medium and evaluated the expression of markers of cementoblast such as bone sialoprotein (BSP), dentin matrix protein 1 (DMP1), and collagen type 1A1 (COL1A1) [20–23]. Osteogenic induction significantly upregulated the expression of BSP and DMP1 but not affected the COL1A1 expression at 3 days in HERS-H1 and HERS-C2 cells (Fig. 3a, b).

The differentiation of HERS cells into cementoblasts was also a process of EMT [7], so we added TGF-β1 into the osteogenic medium to investigate whether TGF-β1 could promote osteogenic differentiation. Compared to osteogenic induction alone, TGF-β1 treatment combined with osteogenic induction significantly upregulated the BSP and DMP1 expression at 7 days and COL1A1 expression at 3 days in HERS-H1 cells (Fig. 3a). In HERS-C2 cells, TGF-β1 treatment combined with osteogenic induction significantly upregulated the BSP and DMP1 expression from 3 days to 7 days, and COL1A1 expression at 7 days (Fig. 3b). Alizarin red S staining showed that more calcium nodules were formed in HERS-C2 cells upon the osteogenic induction compared with the HERS-H1 cells (Fig. 3c). Treatment with TGF-β1 significantly increased the calcium nodule formation especially in HERS-H1 cells (Fig. 3c). The quantification analysis confirmed the significantly higher cementogenic capacity of HERS-C2 cells and TGF-β1 treatment promoted the cementogenic differentiation (Fig. 3d–f).

**Fig. 4:**
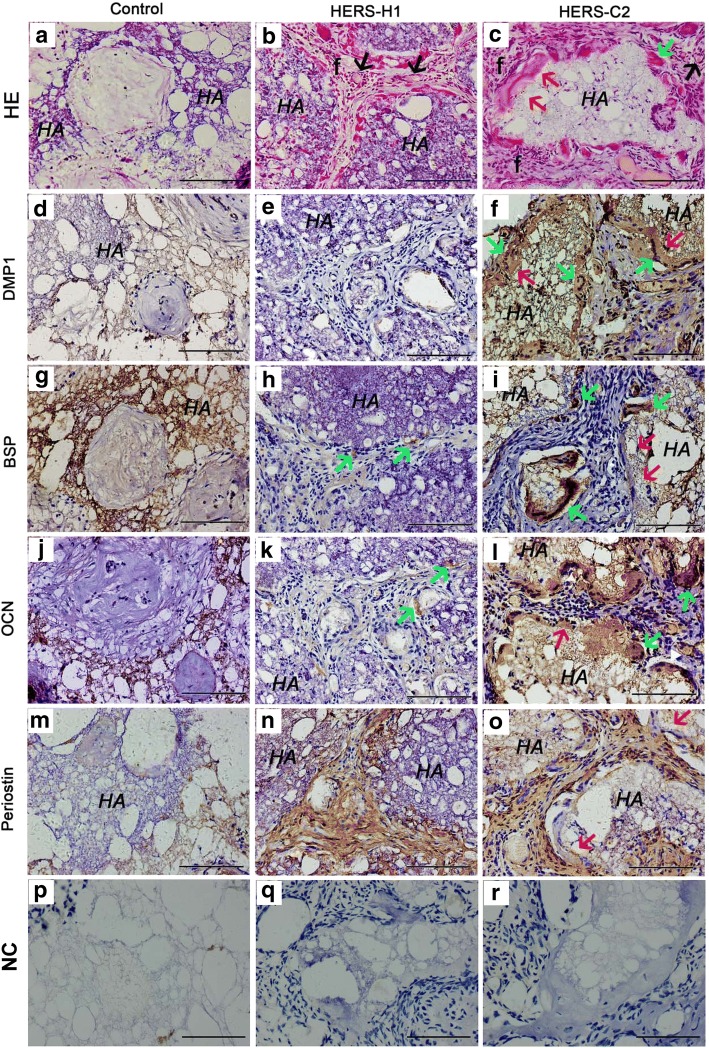
In vivo cementogenesis of HERS-H1 and HERS-C2. **a** After 8 weeks of transplantation, there were few cells grown in the control transplants. **b** Only a dense mass of periodontal ligament fiber-like tissues (**f**) and fibroblast-like cells were found and there was no mineralized structure formed in HERS-H1 transplants. An abundance of blood vessels was formed among the tissues (black arrow). **c** Thin layer of cementum-like tissue (red arrow) and some cementoblast-like cells (green arrow) were generated on the surface of hydroxyapatite/tricalcium phosphate (HA/TCP) particles in HERS-C2 transplants. Periodontal ligament fiber-like tissues (**f**) were observed among the mineralized structure. Immunohistochemistry analysis of the HERS-C2 grafts (**f**, **i**, **l**) showed strong positive staining for DMP1, BSP, and OCN in the cementum-like tissues (red arrow) and the cementoblast-like cells (green arrow), compared to the negative staining with the corresponding antibodies in the control transplants (**d**, **g**, **j**), respectively. In HERS-H1 transplants, **e** DMP1 was totally negative and (**h**, **k**) BSP and OCN were positive in only a few cells (cyan arrow). **m** Periostin was negative in the control grafts. **n**, **o** The fiber-like tissues both in HERS-H1 and HERS-C2 groups were positive for Periostin. **p**, **q**, **r** Negative controls (NC) did not show staining. (HA: HA/TCP particles. Scale bar: 100 μm)

To confirm the capacity of cementogenesis of the two cell lines in vivo, HERS-H1 cells and HERS-C2 cells were separately transplanted into renal capsule of adult SD rats with HA/TCP particles. After 8 weeks transplantation, 3 out of 4 HERS-C2 grafts generated mineralized cementum-like tissue, cementoblast-like cells, and fibrous tissue (Fig. 4c). However, there was no cementum-like tissue formation in all four grafts in HERS-H1 group and only fiber-like structure could be observed (Fig. 4b). In control group, no cementum-like or fiber-like tissues were observed (Fig. 4a). Immunohistochemical staining showed that newly formed tissues of HERS-C2 group were positive expression of DMP1 (Fig. 4f), BSP (Fig. 4i), OCN (Fig. 4l), and Periostin (Fig. 4o). While in HERS-H1 group, the newly formed tissues were negative expression of DMP1 (Fig. 4e), BSP (Fig. 4h), and OCN (Fig. 4k) except for Periostin (Fig. 4n). These results suggested that the two cell lines maintained different biological function. HERS-C2 could differentiate into cementoblasts and generate cementum-like tissue in vivo while HERS-H1 could not.

### Induction of DPC differentiation into odontoblasts by HERS-H1 and HERS-C2 cells

**Fig. 5:**
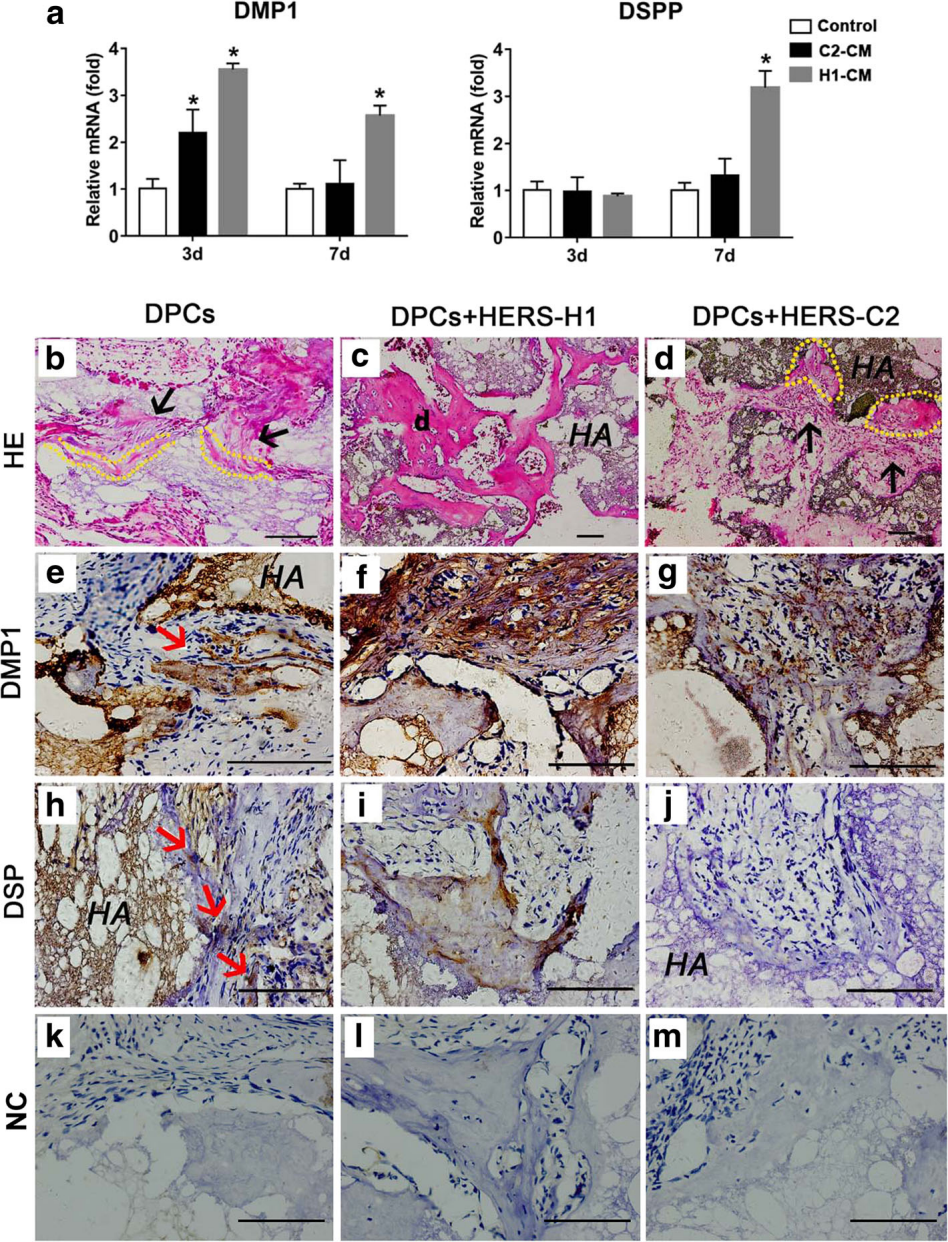
Induction of DPC differentiation into odontoblasts and osteodentin generation by HERS-H1 and HERS-C2 cells. **a** Real-time PCR showed conditioned medium of HERS-H1 or HERS-C2 altered the expression of DMP1 and DSPP in DPCs. **b**, **d** After 8 weeks of transplantation, thin layers of dentin-like tissue (yellow imaginary line) and unmineralized fibrous tissue (black arrow) was formed in the DPCs and HERS-C2 + DPC transplants. **c** A mass of osteodentin (**d**) was generated in HERS-H1 + DPC group. **f**, **i** IHC analysis of HERS-H1 + DPC grafts showed strong positive staining of DMP1 and DSP in the osteodentin and differentiated DPCs. **e**, **h** Only a few cells in the control group showed positive staining for DMP1 and DSP (red arrow). **g**, **j** In HERS-C2 + DPC transplants, a small number of cells showed positive staining for DMP1 while DSP staining was completely negative. **k**, **l**, **m** Negative controls (NC) did not show staining. (*P < 0.05 vs. control group; CM: conditioned medium; Scale bars: 100 μm)

To investigate whether the two HERS cell lines have the capacity to promote the differentiation of the DPCs as primary HERS cells, the DPCs were treated with the conditioned medium (CM) of the two HERS cell lines and the odontoblastic differentiation marker genes such as DMP1 and DSPP were detected. There was a significant increase in expression level of DMP1 in DPCs after treatment with CM of HERS-H1 for 3 days or 7 days, the DSPP expression was also significantly increased after treatment with CM of HERS-H1 for 7 days (Fig. 5a). However, DPCs treated with CM of HERS-C2 did not show a significant increase of DSPP expression either at 3 days or at 7 days (Fig. 5a), and the expression of DMP1 was increased only after treated with CM of HERS-C2 for 3 days but not for 7 days treatment (Fig. 5a). To confirm the induction of odontoblastic differentiation of DPCs of the two cell lines, we mixed HERS-H1 or HERS-C2 cells with DPCs and transplanted them into renal capsule of adult SD rats for 8 weeks. In HERS-H1 combined with the DPC group, abundant osteodentin-like tissue was observed (Fig. 5c) and these newly formed tissue positively expressed DMP1 and DSP (Fig. 5f, i). However, in DPC group or HERS-C2 combined with DPC group, the newly formed osteodentin-like tissue was obviously thinner than that formed in HERS-H1 combined with DPC group (Fig. 5b, c), and the expression of DMP1 and DSP was also obviously weaker than that in HERS-H1 combined with DPC group (Fig. 5e, g, h, and j).

### Gene expression profiles of immortalized HERS cell lines and primary HERS cells

To elucidate the molecular mechanisms responsible for the functional difference between the HERS-H1 and HERS-C2 cells, we conducted gene expression profile microarray on HERS-H1 cells and HERS-C2 cells, and primary HERS cells were included as a control. To assess the validity of the microarray data, real-time quantitative PCR was performed to compare gene expression levels between the cell lines and primary cells. Five genes (Fgf2, Igf1, Integri a1, Ambn, and Amgn) were selected at random from array dataset to validate the microarray data. In all cases, RT-PCR data was consistent with the results of the microarray, suggesting that the dataset obtained from the microarray analysis accurately reflects gene expression differences between HERS cell lines and primary HERS cells (Additional file 4: Figure S5).

**Fig. 6:**
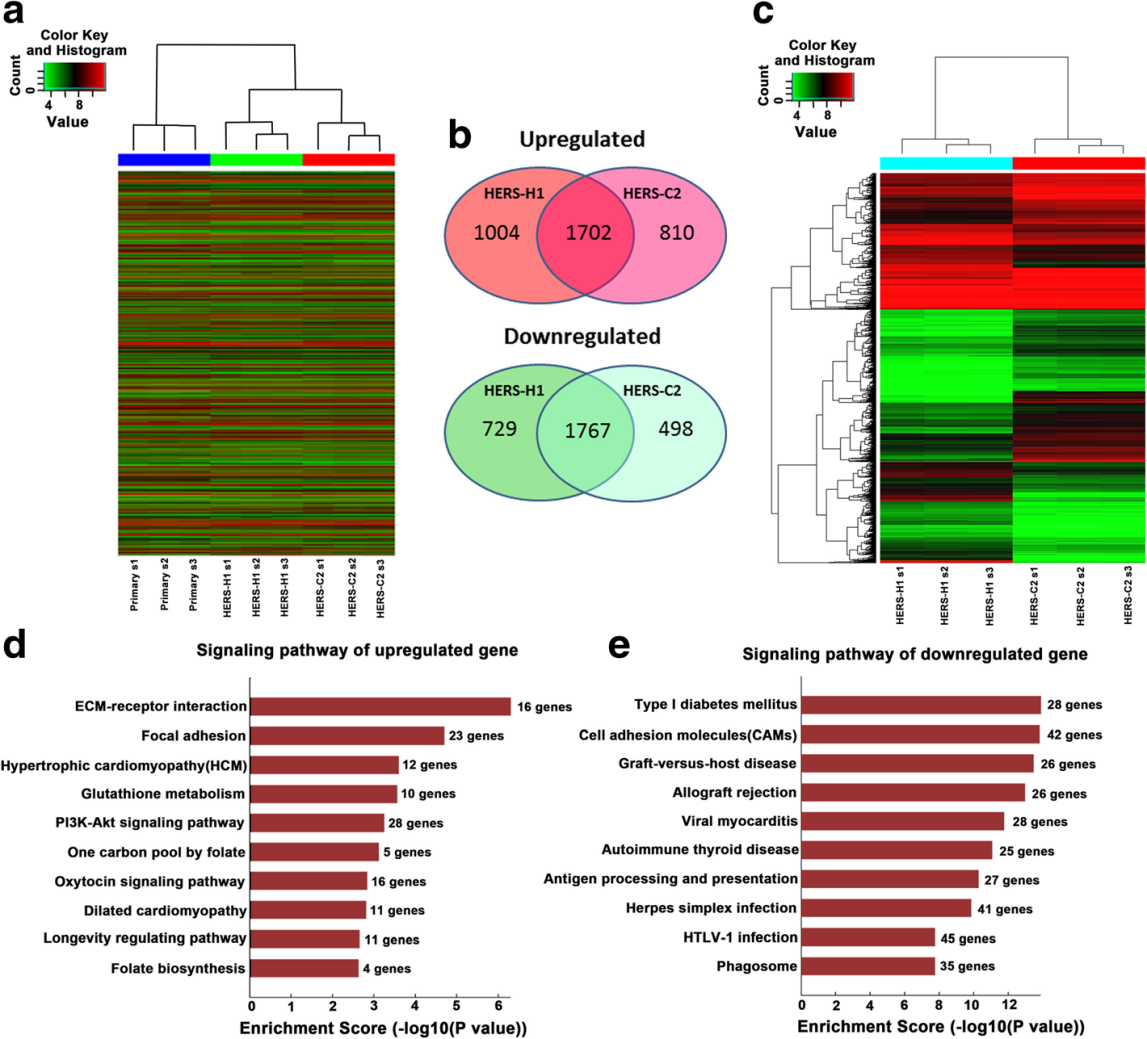
Comparison of gene expression profiles between HERS-H1 and HERS-C2. **a** Heat map showing differential gene expression of primary HERS cells, HERS-H1 cells and HERS-C2 cells. **b** Venn diagram showing common and up- or downregulated genes between primary HERS cells and HERS-H1 cells or HERS-C2 cells. **c** Heat map showing differential gene expression between HERS-H1 cells and HERS-C2 cells. **d** Signaling pathway mapping showing the signaling pathway involved in the upregulated genes in HERS-H1 cells compared to HERS-C2 cells. **e** Signaling pathway mapping showing the signaling pathway involved in the downregulated genes in HERS-H1 cells compared to HERS-C2 cells

We first attempted to identify the gene expression difference between primary HERS cells and HERS-H1 or HERS-C2 cells. Compared to the primary HERS cells, there were 5202 differential expression genes (2706 genes were upregulated and 2496 genes were downregulated) in HERS-H1 cells and 4777 differential expression genes (2512 genes were upregulated and 2265 genes were downregulated) in HERS-C2 cells respectively (Fig. 6a, b). Specifically, 1702 were upregulated and 1767 were downregulated in both HERS-H1 cells and HERS-C2 cells compared to primary HERS cells as shown in the Venn diagram (Fig. 6b). We had validated the expression of a number of identified differential expression genes using RT-PCR to be true positives, confirming that the microarray results were of high quality and reliability (data not shown). Gene Ontology (GO) analysis and pathway mapping revealed that these differential expression genes compared to primary HERS cells were closely related to the PI3K-Akt signaling pathway, Hippo signaling pathway, TGF-β signaling pathway, and so on (Additional file 5: Figure S3).

More importantly, compared to the HERS-C2 cells, there were a total of 834 genes upregulated and 1236 genes downregulated in HERS-H1 cells (Fig. 6c). Based on Gene Ontology (GO) analysis, the upregulated genes were mostly related to the tissue development and ossification (Additional file 6: Figure S4a), and the down genes mostly related to the cell differentiation (Additional file 6: Figure S4b). Moreover, function/pathway analysis via Kyoto Encyclopedia of Genes and Genomes (KEGG) of the differential expression genes reveals that many upregulated genes were associated with signaling pathways for ECM-receptor interaction, focal adhesion, and PI3K-AKT signaling pathway (Fig. 6d); on the other hand, many downregulated genes were related to cell adhesion molecules (Fig. 6e).

## Discussion

During the development of the tooth root, HERS cells occupy a central position in guiding tooth root formation by differentiation into cementoblasts through EMT and induction of odontoblast differentiation through EMI. Previous studies mainly investigated the functions and mechanisms of HERS cells in tooth root development [6, 24]. Based on the mechanism of tooth root development, we focus on the application of HERS cells in tooth root regeneration. However, HERS cells are a temporary structure during the tooth development [25], and limited availability and lifespan of primary HERS cells poses a significant barrier to biological investigation and therapeutic use of tooth tissue engineering. In this study, we established two immortalized non-tumorigenic cell lines HERS-H1 and HERS-C2 deriving from Hertwig’s epithelial root sheath for further research and provided a new cell resource for studies on tooth tissue engineering.

The cell lines should be able to proliferate easily in culture while preserving their functional phenotype. In this study, results on cellular morphology, epithelial or mesenchymal-associated gene expression, and response to TGF-β1 treatment have shown that HERS-H1 and HERS-C2 cells retain characteristic of primary HERS cells. Based on the gene expression profiles and pathway mapping, we found that, in immortalized HERS-H1 or HERS-C2 cells, the PI3K-Akt signaling pathway was significantly upregulated and Hippo and TGF-β signaling pathways were significantly downregulated. Previous studies showed that PI3K-Akt signaling pathway and Hippo signaling pathway were closely related to the survival, growth, and proliferation of several of normal stem cells and cancer cells [26–29]. In tooth epithelial stem cells, PI3K-Akt and Hippo signaling pathways were also critical for the cell proliferation and differentiation [30–32]. Moreover, compared to the primary HERS cells, TGF-β signaling pathway was downregulated both in HERS-H1 and HERS-C2 cells. It was confirmed that TGF-β signaling, in HERS cells, relied on a Smad-dependent mechanism in regulating Nfic expression via Shh signaling to control root development [6, 33]. During the tooth root development, TGF-β signaling triggered HERS cells differentiating into cementoblast-like cells via EMT [7, 8, 34]. In HERS-H1 and HERS-C2 cell lines established in our study, TGF-β signaling was downregulated which might be responsible for the inhibition of HERS cell differentiation by inhibited EMT. When the cell lines were treated with exogenous TGF-β1, the expression level of mesenchymal markers and transcriptional factors was upregulated, indicating that EMT program was reactivated. Moreover, exogenous TGF-β1 treatment also promoted the differentiation of HERS cell lines in vitro and in vivo. In addition, to evaluate the safety of the cell lines for tooth root regeneration, we tested whether the cell lines could transform into tumor cells in vivo. After 4 weeks of transplantation, xenograft tumor formation was found in SCC-25 group but not in HERS-C2 and HERS-H1 cells. These results indicate that HERS-C2 and HERS-H1 cell lines may provide good models to study the function of HERS cells and may be new suitable cell resources of tooth root regeneration.

HERS cells were constituted by a heterogeneous cell population [35]. In this study, we surprisingly found the two cell lines had unique features; one of them had the ability to differentiation into cementum-like cells, while the other had the ability to induce the odontoblastic differentiation of dental mesenchymal cells. As previous studies identified, HERS cells were capable of forming mineralized matrix and expressing osteogenic genes when cultured under osteo-media [15, 36]. Our results proved that osteo-inductive condition and the combined treatment of TGF-β1 could trigger the two cell lines differentiating into cementoblasts. In vivo transplantation showed that in vitro expanded HERS-C2 cells generated cementum characterized by a layer of aligned cementum-like structure. Nevertheless, HERS-H1 cells could only generate fibrous tissue without mineralized structure formation, suggesting that only a subset of HERS cells carried the capacity of forming cementum-like tissue.

If the continuity of HERS is disturbed, the differentiation of root odontoblasts is compromised [9]. Thus, another characteristic of HERS cell heterogeneity is induction of odontoblast differentiation. Some studies implied that the HERS could induce the differentiation of the odontoblasts and suggested that HERS functions as a signaling center to guide root formation [6, 37]. Our in vitro studies proved that the CM of HERS-H1 could promote the differentiation of DPCs. To investigate whether the cell lines could induce the formation of dentin in vivo, we also transplanted HERS-H1 and DPCs with HA/TCP into renal capsule of adult SD rats. After 8 weeks of transplantation, we found a large amount of dentin-like tissue formed and the DSP and DMP1 expression was accelerated by the combination of HERS-H1, indicating that only HERS-H1 possessed the capacity of promoting the differentiation of DPCs and this cell line could be used to regenerate dentin and reconstruct tooth root as well as the alveolar bone.

In present study, we established two novel cell lines derived from Hertwig’s epithelial root sheath, which represented two key features of HERS cells during the tooth root development. In order to clarify the mechanisms contributing to the difference between these two cell lines, we compared the gene expression profiles and found that a serious of signaling pathways was involved such as ECM-receptor interaction signaling. The extracellular matrix (ECM) is defined by the composite accumulation of structural and functional molecules that are secreted by cells of all tissues and arranged in a tissue-specific 3D ultrastructure [38]. ECM consists of a complex mixture of structural and functional macromolecules including glycosaminoglycans and fibrous proteins (collagen, elastin, fibronection, and lammin). Specific interactions between cells and the ECM are mediated by transmembrane molecules integrins [39]. The expression of integrin showed a cell type-specific pattern and the integrin subunits determined which ECM substrates bind to which cells, and ultimately influences cellular phenotype and function [40]. The ECM regulates numerous biological processes, including angiogenesis, innervation, and stem cell differentiation. In fact, previous study has demonstrated that ECM is essential for tissue architecture and development in bone and tooth [41]. However, in dental-derived stem cells especially HERS cells, the function of ECM has not received sufficient attention. In our data, the genes such as COL6A1, LAMA3, LAMA2, ITGA10, TGA4, and ITGA7 related to ECM-receptor interactions were observed to be upregulated and signaling pathway enrichment analysis showed that ECM-receptor interactions were significantly upregulated in HERS-H1 cells compared to HERS-C2 cells. ECM is not only a product of cellular activity but is also a regulator of cellular activity [39]. These higher expression genes involved in ECM-receptor interactions might mediate the intercellular interaction and communication between epithelia and mesenchyme. These also might be a major reason for the HERS-H1 cells showing more potential to promote the differentiation of DPCs than HERS-C2 cells. To thoroughly elucidate the specific mechanism, further researches are still in need. The two cell lines possess two functions of HERS epithelial cells respectively: epithelial-mesenchymal transition and epithelial-mesenchymal interaction, which is essential for tooth root formation. As for the tooth root tissue regeneration, combination of the two cell lines and dental mesenchyme might imitate the natural process of tooth root development. Compared with the single mesenchymal stem cell-based approach of tooth tissue regeneration, our cell lines derived from epithelia combined with mesenchymal cell-based method could generate more kinds of tooth tissues and provide an inductive microenvironment that mimics the natural niche for cell growth and differentiation.

## Conclusion

This study reports two novel immortalized epithelial cell lines which represent two key features of HERS cells during the tooth root development for the first time. Both two cell lines did not induce tumorigenesis in vivo and proved to be biologically safe for implantation. HERS-C2 could differentiate into cementoblasts which may produce cementum. HERS-H1 could promote the differentiation and dentin formation of DPCs. The in vitro and in vivo experiments also confirm that HERS cells are of heterogeneity and suggest that the two cell lines provide substitutes for primary cells in biologically relevant applications in the fields of cell biology, developmental biology, and tooth tissue engineering, which constitute a novel and promising approach for future clinical dental therapy.

## Additional files

Additional file 1: **Table S1.** Primer sequences used for quantitative real-time PCR. (DOCX 15 kb)

Additional file 2: **Figure S1.** The examination of tumorigenicity of two immortalized cell lines. (A) Macroscopic appearance and (B) tissue sections of HE stain showed injection of primary HERS cells, HERS-C2 cells, HERS-H1 cells into nude mice did not formation tumor after 4 weeks; tumor formation was observed in positive control group SCC-25 cells (red imaginary line). Scale bars: 200 μm. (TIF 12083 kb)

Additional file 3: **Figure S2.** Seven days induction by TGF-β1 of HERS-H1 cells. The expression level of E-cadherin was downregulated and the level of vimentin, N-cadherin and snail1 upregulated examined by real-time RT-PCR. (*P < 0.05; **P < 0.01 vs. control). (TIF 587 kb)

Additional file 4: **Figure S5.** Confirmation of microarray data using real-time RT-PCR. Real-time RT-PCR was carried out to validate the array results. Total RNA from three independent cultured cells were used for the analysis. Triplicate assays were performed from each RNA sample. Data are normalized using GAPDH as an endogenous control for RNA input. (TIF 5232 kb)

Additional file 5: **Figure S3.** Signaling pathway mapping of HERS-H1 cells and HERS-C2 cells. (A) Signaling pathway of upregulated genes of HERS-H1 vs. primary HERS cells. (B) Signaling pathway of downregulated genes of HERS-H1 vs. primary HERS cells. (C) Signaling pathway of upregulated genes of HERS-C2 vs. primary HERS cells. (D) Signaling pathway of downregulated genes of HERS-C2 vs. primary HERS cells. (TIF 1013 kb)

Additional file 6: **Figure S4.** Go analysis of the differential expression genes. (A) Function analysis of the upregulated genes of HERS-H1 cells compared to HERS-C2 cells was conducted via GO analysis. (B) Function analysis of the downregulated genes of HERS-H1 cells compared to HERS-C2 cells was conducted via GO analysis. (TIF 602 kb)

## Abbreviations

CM: Conditioned medium
DPCs: Dental papilla cells
EMI: Epithelial–mesenchymal interaction
EMT: Epithelial–mesenchymal transition
HA: Hydroxyapatite/tricalcium phosphate
HERS: Hertwig’s epithelial root sheath
