# Supplementary material for: Development of immortalized Hertwig’s epithelial root sheath cell lines for cementum and dentin regeneration
Source: Stem Cell Res Ther. 2019 Jan 3;10:3. doi: 10.1186/s13287-018-1106-8 (PMC6319004; doi:10.1186/s13287-018-1106-8)
Supplement: Supplementary file 1 — Table S1. Primer sequences used for quantitative real-time PCR. (DOCX 15 kb) [file 13287_2018_1106_MOESM1_ESM.docx]

**Table S1**. Primer sequences used for quantitative real time-PCR

| Gene | Primer sequence |
| --- | --- |
| E-cadherin  Vimentin  N-cadherin  Twist1  Snail1  Zeb1  BSP  DMP1  Col1A1  bFGF  Igf1  Interrin a1  Notch1  Ambn  Amgn  GAPDH | Forward:5’-TCCTGCTCCTACTGTTTCTACGG-3’  Reverse:5’-TCTTCTTCTCCACCTCCCTCTTC-3’  Forward:5’-CCCTGAACCTGAGAGAAACTAACC-3’  Reverse: 5’-GTCATCGTGGTGCTGAGAAGTC-3’  Forward: 5’-ATGCTGACCACTCTCACTGCTC-3’  Reverse: 5’-ACATTTGGCGACTCTCTGTCC-3’  Forward: 5’- ACCCTCACACCTCTGCATTC-3’  Reverse: 5’- CAGTTTGATCCCAGCGTTTT-3’  Forward: 5’- GAGGACAGTGGCAAAAGCTC-3’  Reverse: 5’- TCGGATGTGCATCTTCAGAG-3’  Forward: 5’- AGGCAAATGGTTGAAACTGG-3’  Reverse: 5’- TGCATCTGGTGTTCCATTGT-3’  Forward: 5’-GAAAGAGCAGCACGGTTGAGTA-3’  Reverse: 5’-CGTCCTCATAAGCTCGGTAAGTG-3’  Forward: 5’-CCGATAAGGAGGAGGATGAAGA-3’  Reverse: 5’-ACTGGACTGTGTGGTGTCTGC-3’  Forward: 5’-TCTGACTGGAAGAGCGGAGAG-3’  Reverse: 5’-GAGTGGGGAACACACAGGTCT-3’  Forward: 5’-GAACCGGTACCTGGCTATGA-3’  Reverse: 5’-CAGTTCGTTTCAGTGCCACA-3’  Forward: 5’-TCTCCTAGTCCCTGCCTCTT-3’  Reverse: 5’-TCTGTGAAGGAAGCGGCTTA-3’  Forward: 5’-ACGAACACATTCCCTTTGCC-3’  Reverse: 5’-TGTTGTACGCACTGTCTCCT-3’  Forward: 5’- AGCCTCGATTTTCCTGGTCA-3’  Reverse: 5’- AAGTTTCCAGCCTCCTCCTC-3’  Forward: 5’- AGGACTTCTTGCTTTCCCCA-3’  Reverse: 5’- TTTGTTTCCAGGCATCGGTG-3’  Forward: 5’- TCAGCCAAACATCCCTCCAT-3’  Reverse: 5’- CTTGGTCTTGTCTGTCGCTG-3’  Forward: 5’- TATGACTCTACCCACGGCAAG-3’  Reverse: 5’- TACTCAGCACCAGCATCACC-3’ |
| Gene | Primer sequence |
