# Supplementary figures and images for: Development of immortalized Hertwig’s epithelial root sheath cell lines for cementum and dentin regeneration
Source: Stem Cell Res Ther. 2019 Jan 3;10:3. doi: 10.1186/s13287-018-1106-8 (PMC6319004; doi:10.1186/s13287-018-1106-8)

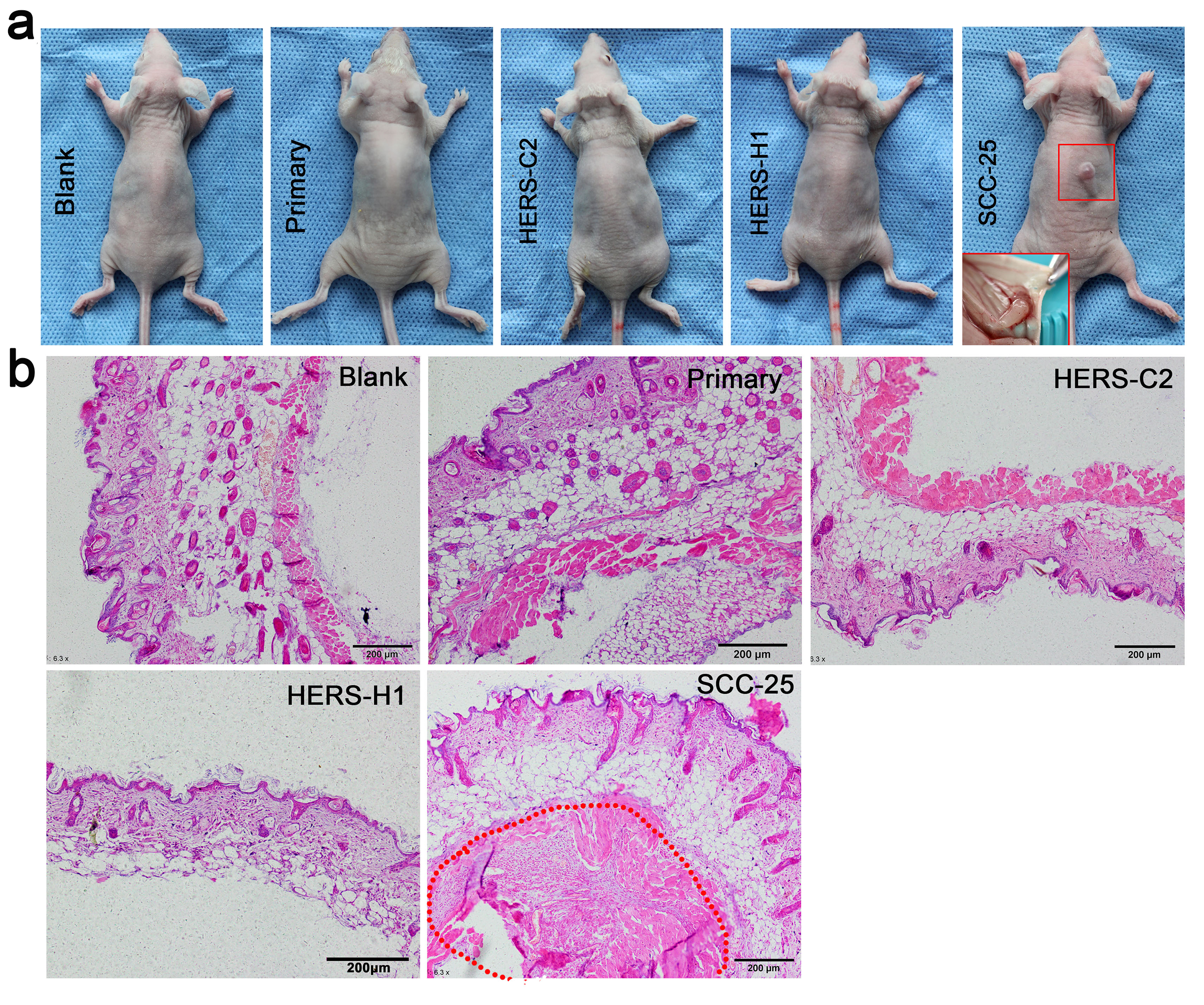

Supplement: Supplementary file 2 — Figure S1. The examination of tumorigenicity of two immortalized cell lines. (A) Macroscopic appearance and (B) tissue sections of HE stain showed injection of primary HERS cells, HERS-C2 cells, HERS-H1 cells into nude mice did not formation tumor after 4 weeks; tumor formation was observed in positive control group SCC-25 cells (red imaginary line). Scale bars: 200 μm. (TIF 12083 kb) [file 13287_2018_1106_MOESM2_ESM.tif]

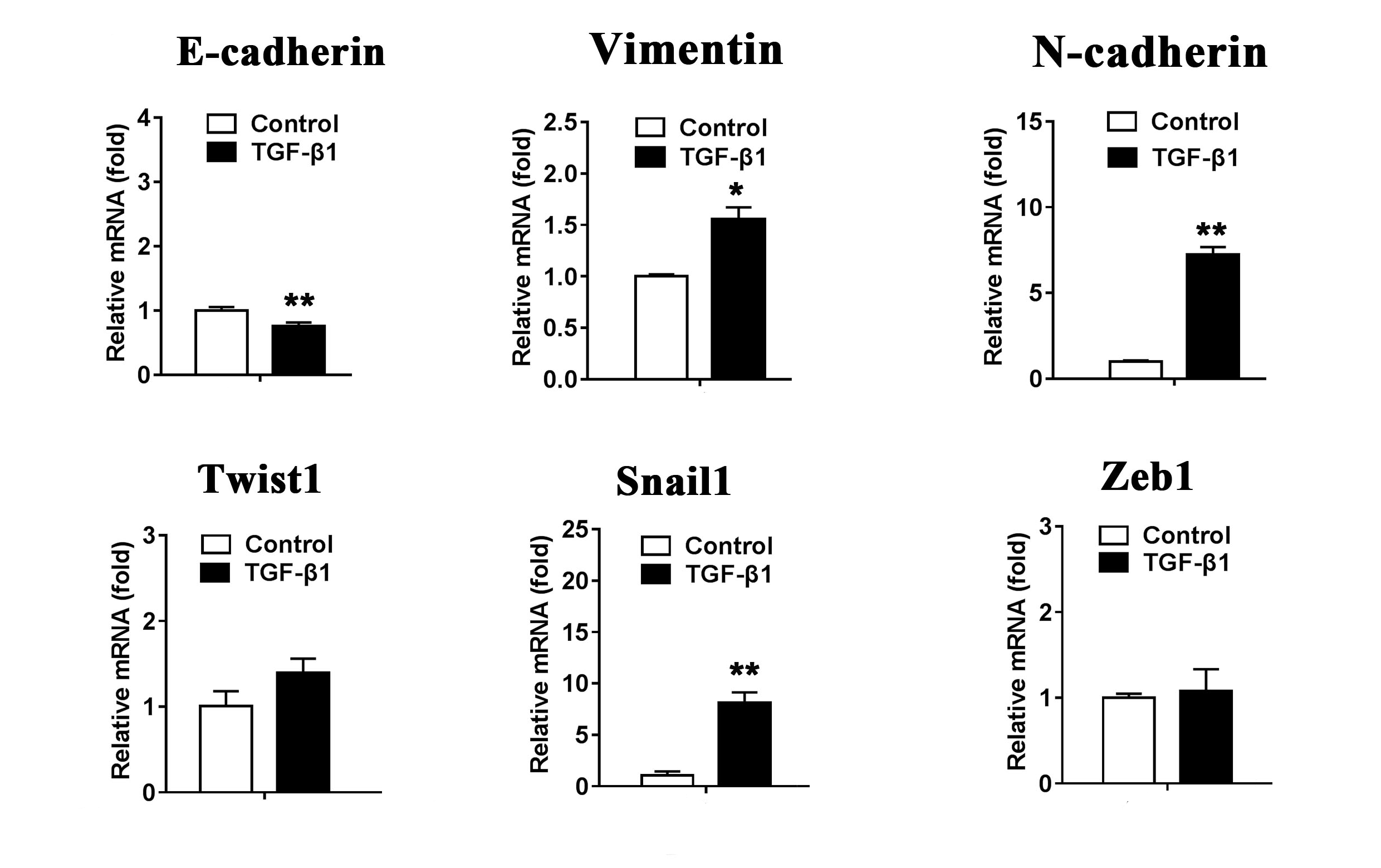

Supplement: Supplementary file 3 — Figure S2. Seven days induction by TGF-β1 of HERS-H1 cells. The expression level of E-cadherin was downregulated and the level of vimentin, N-cadherin and snail1 upregulated examined by real-time RT-PCR. (*P < 0.05; **P < 0.01 vs. control). (TIF 587 kb) [file 13287_2018_1106_MOESM3_ESM.tif]

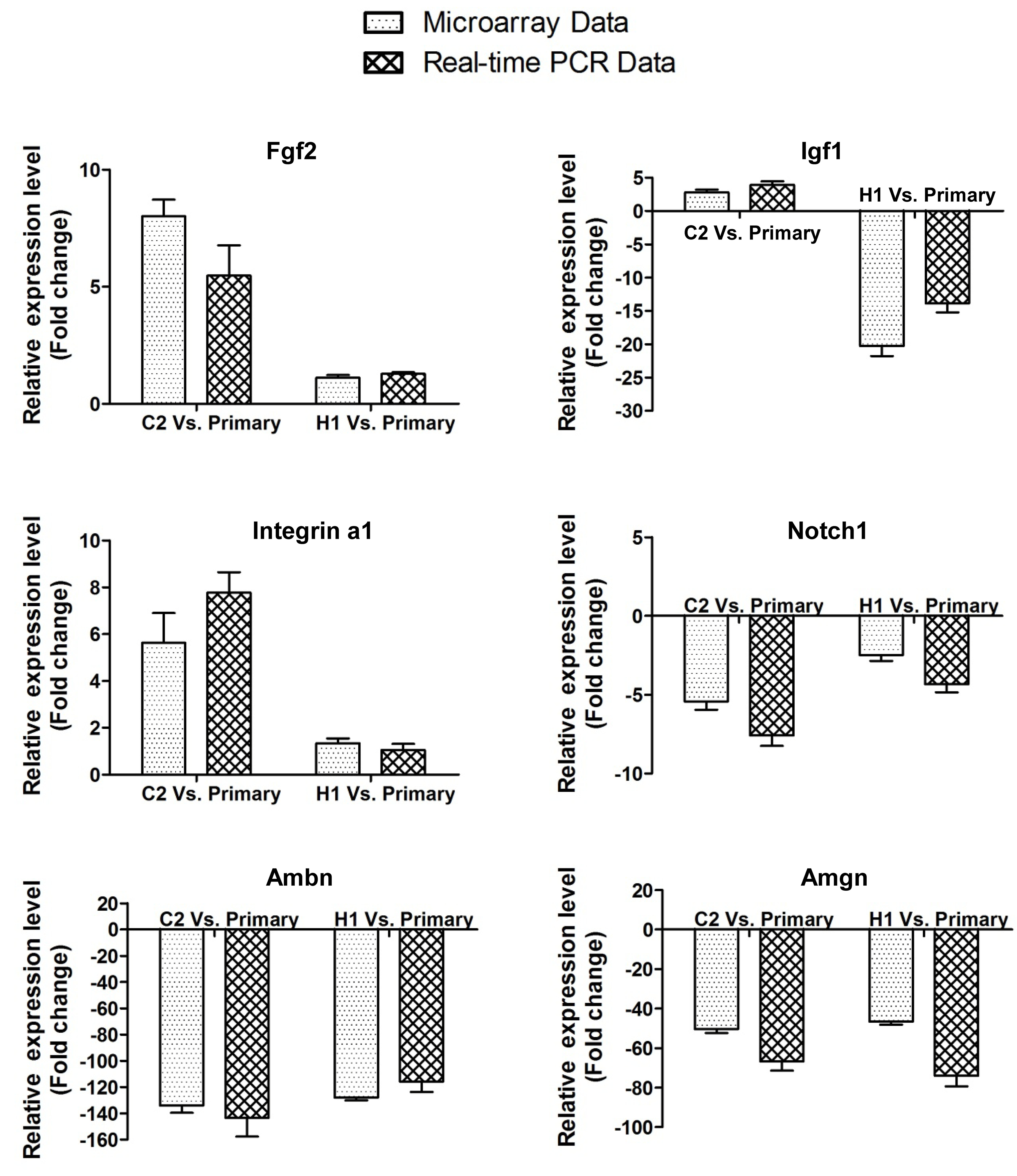

Supplement: Supplementary file 4 — Figure S5. Confirmation of microarray data using real-time RT-PCR. Real-time RT-PCR was carried out to validate the array results. Total RNA from three independent cultured cells were used for the analysis. Triplicate assays were performed from each RNA sample. Data are normalized using GAPDH as an endogenous control for RNA input. (TIF 5232 kb) [file 13287_2018_1106_MOESM4_ESM.tif]

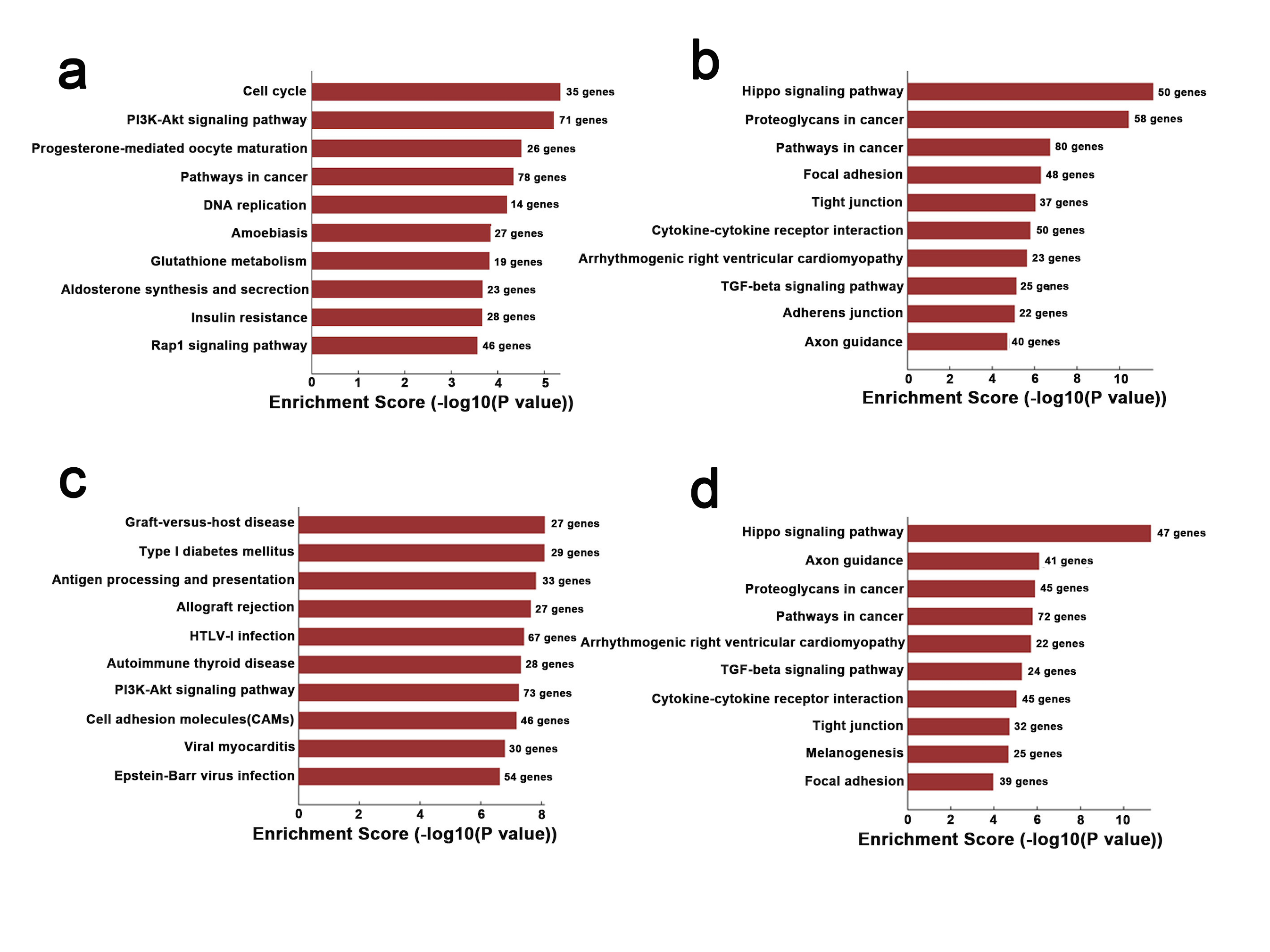

Supplement: Supplementary file 5 — Figure S3. Signaling pathway mapping of HERS-H1 cells and HERS-C2 cells. (A) Signaling pathway of upregulated genes of HERS-H1 vs. primary HERS cells. (B) Signaling pathway of downregulated genes of HERS-H1 vs. primary HERS cells. (C) Signaling pathway of upregulated genes of HERS-C2 vs. primary HERS cells. (D) Signaling pathway of downregulated genes of HERS-C2 vs. primary HERS cells. (TIF 1013 kb) [file 13287_2018_1106_MOESM5_ESM.tif]

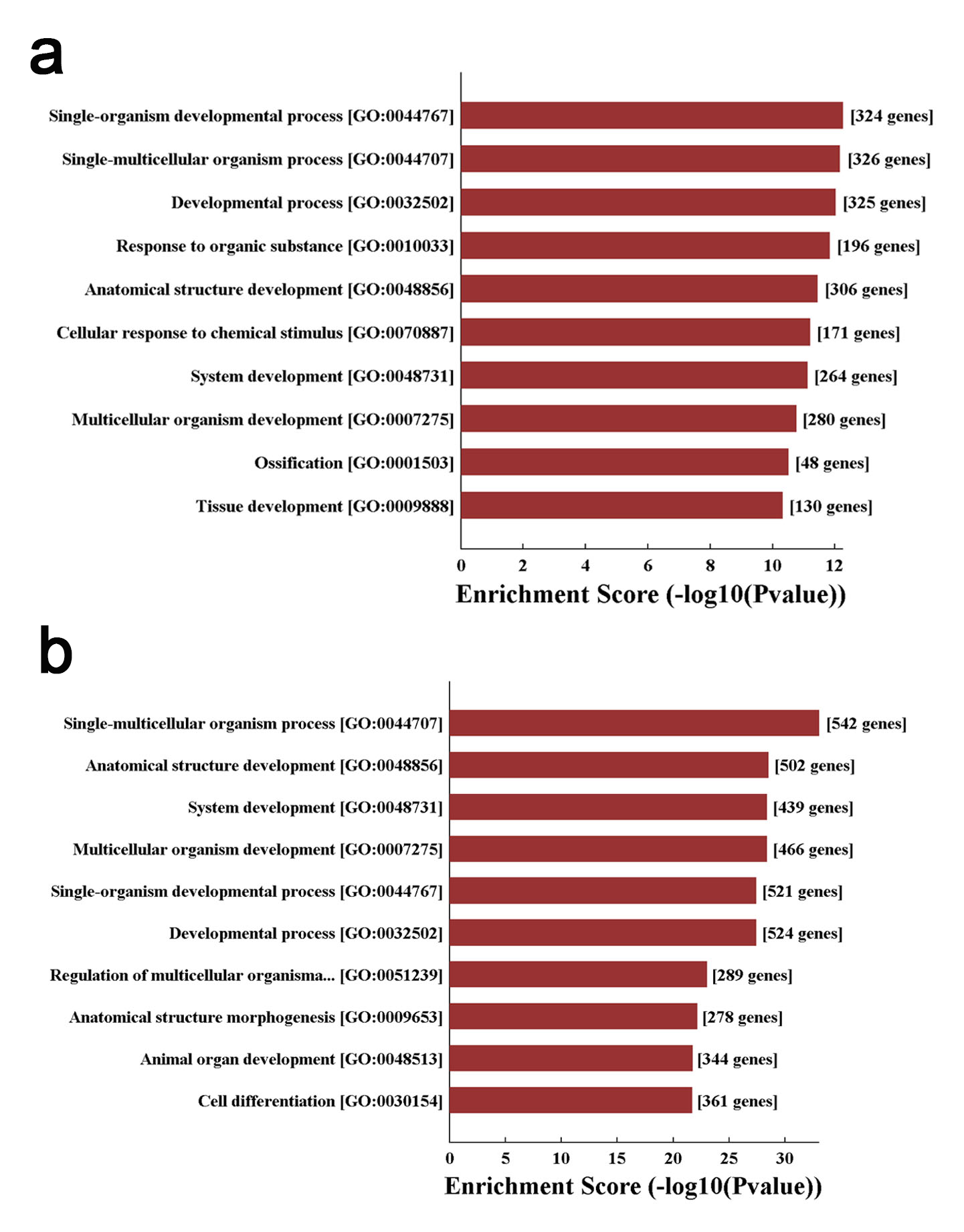

Supplement: Supplementary file 6 — Figure S4. Go analysis of the differential expression genes. (A) Function analysis of the upregulated genes of HERS-H1 cells compared to HERS-C2 cells was conducted via GO analysis. (B) Function analysis of the downregulated genes of HERS-H1 cells compared to HERS-C2 cells was conducted via GO analysis. (TIF 602 kb) [file 13287_2018_1106_MOESM6_ESM.tif]
